# A case report of ADMIO type 1 caused by a *de novo STAT3* gain-of-function mutation

**DOI:** 10.3389/fimmu.2026.1824739

**Published:** 2026-08-28

**Authors:** Lan Huang, Yang Yue, Yuanhui Zhu, Houhua Hao, Fengjun Guan

**Affiliations:** Department of Pediatric Nephrology and Rheumatology Immunology, Affiliated Hospital of Xuzhou Medical University, Xuzhou, Jiangsu, China

**Keywords:** ADMIO type 1, STAT3 GOF, arthritis, autoimmune disease, HSP, adalimumab, JAK inhibitor

## Abstract

Infantile-onset Multisystem Autoimmune Disease type 1(ADMIO, 1) is a rare hereditary autoimmune disorder primarily caused by *STAT3* gain-of-function (GOF) mutations. This article presents a case of ADMIO type 1 resulting from a *de novo STAT3* GOF mutation. The subject is a male infant who presented with a recurrent rash on both lower limbs. He had a 9-month history of vasculitis, initially diagnosed as Henoch-Schönlein purpura(HSP), with recurrent purpura, ankle arthritis, livedo reticularis, and chronic diarrhea. Diagnosis was confirmed via whole-exome sequencing identifying a heterozygous *STAT3* mutation (c.2107G>A, p.A703T). Symptoms recurred frequently during corticosteroid tapering. Due to his genetic findings, targeted therapy was required. Initial treatment with tocilizumab provided short-lived benefit. Following recurrent lymphadenitis and polyarthritis, adalimumab, tofacitinib, ruxolitinib emerged as a key anti-inflammatory intervention to treat the disease. This case enriches the rare disease database and helps raise clinicians’ awareness of and attention to ADMIO type 1 disease.

## 1 Introduction

Henoch-Schönlein purpura is an allergic vasculitis that affects the small arteries and capillaries of the skin and other organs. Its main clinical manifestations include non-thrombocytopenic purpura, arthritis or arthralgia, abdominal pain, gastrointestinal bleeding, and renal involvement. It is also known as Henoch-Schönlein purpura or IgA vasculitis. ADMIO type 1(Infantile-onset Multisystem Autoimmune Disease, 1), also known as *STAT3*-related early-onset multisystem autoimmune disease and *STAT3* GOF sydrome, is a rare autosomal dominant disorder primarily caused by GOF mutations in the *STAT3* gene. Its prevalence is less than 1 in 1,000,000. ADMIO type 1 typically presents in infancy or early childhood with various autoimmune manifestations and immune dysregulation (1–9). Most affected children exhibit lymphadenopathy, autoimmune cytopenia, multi-organ autoimmunity affecting the lungs, gastrointestinal tract, liver, or endocrine system, recurrent infections, and short stature (**Table 1**). Laboratory and imaging evaluations reveal thyroid dysfunction, leukopenia, erythrocytopenia, thrombocytopenia, positive Coombs tests, positive autoantibodies, positive infection markers, aberrant T-cell subset differentiation(e.g., increased double-negative T cells), reduced IgE levels, and positive findings on lung, lymph node, or joint ultrasonography. The characteristics of ADMIO type 1 include lymphoproliferation and early-onset solid organ autoimmune diseases. Elevated serum inflammatory cytokine levels, autoimmune antibody abnormalities, and whole-exome sequencing variations are common. Symptoms may appear sequentially, with the respiratory system, endocrine system, and immune system being the most commonly involved systems at onset, while involvement of the circulatory system is rare. Asymptomatic genetic mutations are also possible, and there is a wide range of phenotypic variability.

**Table 1:** Main clinical manifestations of ADMIO type 1.

System	Clinical Manifestations	Symptoms of the patient
Growth and Development	Short stature, growth retardation	+
Head and neck	Dental abnormalities	-
Respiratory System	Interstitial pneumonia	+
Digestive System	Autoimmune enteritis, celiac disease, autoimmune hepatitis, celiac disease	+
Skeletal System	Arthritis	+
Skin, Nails and Hair	Eczema, dermatitis	+
Endocrine System	Type 1 diabetes mellitus, hypothyroidism (in some patients), delayed puberty	-
Hematological System	Autoimmune cytopenia, autoimmune hemolytic, autoimmune thrombocytopenia, lymphadenopathy, hepatosplenomegaly, leukemia, lymphoma	+
Immunological System	Positive serum autoantibodies, recurrent infections, altered T-cell regulation, hypoglobulinemia, increased number of double-negative T cells (CD4^-^, CD8^-^), arthritis, ocular inflammation	+
Urinary System	Chronic renal impairment, kidney stones, renal tubular dysplasia	-
Others	Onset in early childhood, variable presentation, mostly caused by *de novo* mutations	+

STAT3 GoF disease is inherited in an autosomal dominant manner. Previous reports of STAT3 GoF mutations confirms the variable expressivity of this rare condition with variable phenotypic severity and variable age of onset of the various manifestations, challenging the notion of a unique phenotypic pattern for this disease. Milner JD (10) documented three patients (11) with the c.2107G>A, p.Ala703Thr mutation, who came from one family, demonstrating autosomal dominant familial inheritance. Nevertheless, the constellation of phenotypes observed in this case is distinctive (12–21). Regarding other mutations associated with this disease, Leiding JW (22) and colleagues have identified 191 cases to date, in which the severity of symptoms correlates with the structural domain of *STAT3* affected by the mutation, such as c.2144C>A, p.P715Q (presenting with leukocytosis). Herein, we report a patient with a heterozygous missense *STAT3* mutation associated with Henoch-Schönlein purpura and slowly progressive polyarthritis.

## 2 Case presentation

We report a case of an 18-month-old boy(6years old now) with a history of dermatitis and vasculitis, presenting with recurrent purpura of the lower extremities, chronic diarrhea, recurrent upper respiratory tract infections, and growth retardation. The physical examination reveals scattered purpura and ecchymosis on both lower extremities, exhibiting a color gradient from light red to dark red, whose lesions are roundish in shape. Additionally, livedo reticularis and pitting edema are present on the dorsum of both feet and ankle joints. No family history of similar conditions was noted. After excluding infectious etiologies (CMV, EBV, HIV, MP, RSV, AdV, FluA-B, PIV1-3), he was initially diagnosed with HSP and diarrheal disease. Based on the early-onset Henoch-Schönlein purpura accompanied by recurrent livedo reticularis, we first performed whole-exome sequencing(When he was 1 year old). The results allowed us to exclude autoimmune diseases caused by other genes, such as *STAT1* GOF, *CTLA4/LRBA* deficiency, and APLS, and *in vitro* cellular assays(Homology Modeling of STAT3 protein structure prediction and Luciferase reporter assay) ruled out loss-of-function (LOF) mutations in the STAT3 gene. In addition to the purpura and swelling in the ankle joints, joint ultrasound, MRI, and arthrocentesis revealed progressive inflammatory changes in the knee joints, with subsequent involvement of the hip joints. Bilateral knee joint effusion, arthritis, and synovial thickening were particularly prominent. Arthrocentesis of the knee joint cavity showed sterile inflammation. Routine analysis, biochemical tests, and routine bacterial cultures of the joint fluid revealed no significant abnormalities. Infectious causes such as febrile arthritis, joint trauma, and acute osteomyelitis were excluded.

His parents were both wild-type, indicating a *de novo* mutation in the patient. Notably, joint symptoms appeared approximately two years after the genetic diagnosis, with recurrent relapses during glucocorticoid tapering and withdrawal. Joint examination revealed limited range of motion, restricted ambulation with antalgic gait, and a weakly positive ballottement test. The patient exhibited increased cytokine levels(IL-1B, IL-5, IL-8, IL-12, INF-γ, TNF-α), which arrived up to 10 times of normal level. Joint ultrasound showed joint effusion and synovial thickening, with fluid accumulation in the suprapatellar bursa. Joint MRI revealed synovial thickening, consistent with arthritis. At 4 years and 7 months of age, arthrocentesis was performed, and the findings were consistent with sterile arthritis. Specifically, the patient developed arthritis without apparent triggers, involving two or more joints. Rheumatoid arthritis and juvenile idiopathic arthritis were excluded. Given the severity of arthritis leading to activity limitation and significantly elevated cytokine levels and ultrasound results revealed inguinal and cervical lymphadenopathy in the patient as well as chest CT suggested interstitial pneumonia, autoimmune disease was considered the underlying cause (23, 24).

**Figure 1:**
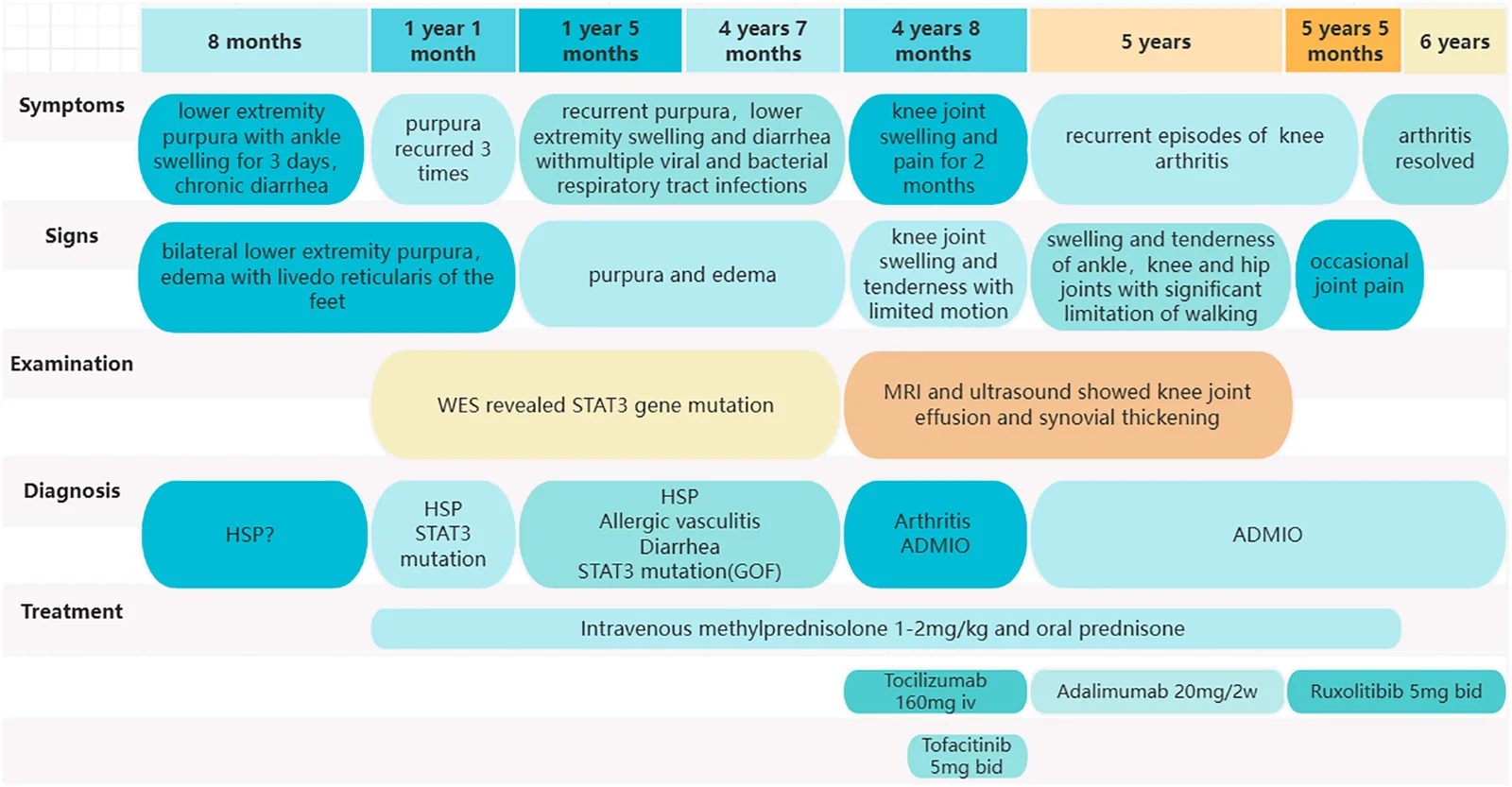
Overview of clinical history and management.

Between the ages of 3 years and 4 years and 3 months, the child experienced approximately 5 relapses of purpura, complicated by bacterial upper respiratory tract infection and vasculitis. 5 months later, he began to develop knee joint effusion, accompanied by arthritis of the ankle and hip joints. During the periods of glucocorticoid use, dose tapering, maintenance, or withdrawal, his polyarthritis recurred repeatedly and severely affected his ability to walk. Therefore, he received his first dose of tocilizumab 160 mg intravenously. At 4 years and 8 months, he was diagnosed with ADMIO type 1. Oral tofacitinib was initiated at a dose of 5 mg/2.5 mg twice daily. After 3 months of symptom relief, he experienced a refractory recurrence of arthritis at the age of 5 years, so the treatment was switched to adalimumab 20 mg subcutaneously/2w. After a period of symptom relief, the symptoms rebounded. Targeted therapy was switched for the third time to ruxolitinib at the age of 5 years and 5 months. He has received regular ruxolitinib treatment for 11 months (**Figure 1**).

**Figure 2:**
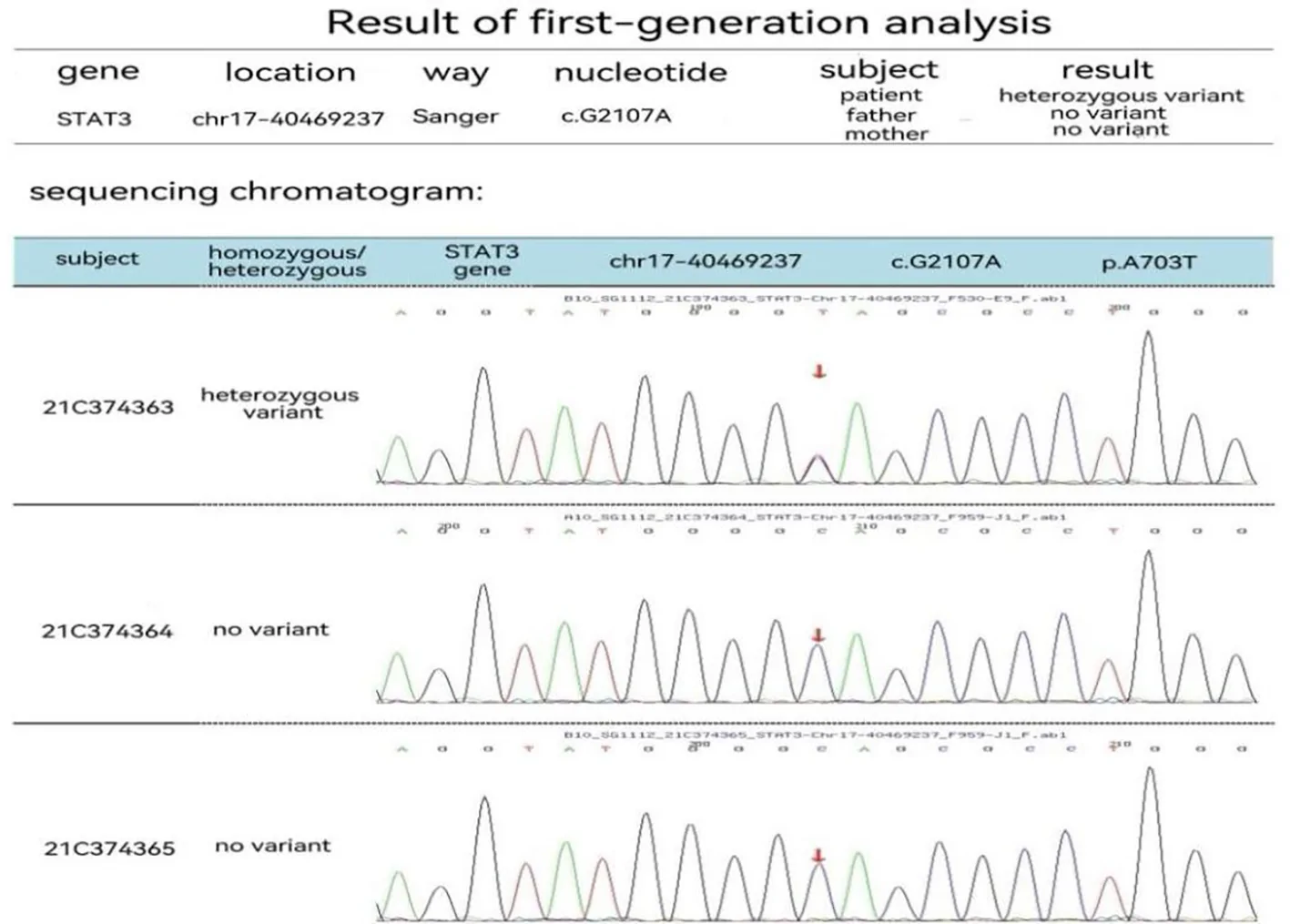
Heterozygous mutation of STAT3 gene on chromosome 17(arrow).

Whole-exome sequencing (WES) (**Figure 2**) identified a heterozygous *STAT3* missense variant of the patient’s, a gene associated with autoinflammatory disorders. According to the ACMG/AMP guidelines, this ariant was classified as likely pathogenic. According to 2015 guidelines criteria, it shows:Genomic location: Chr17: 40469237G>A (GRCh37) {or g.40469237G>A(17q21.2)};cDNA (NM_139276.2: exon22): c.2107G>A;Protein Change: p.Ala703Thr (p.A703T);DbSNP(rsID):869312894;Mutation type: heterozygous missense mutation. The classification as likely pathogenicity is supported by the following items:reported cases of the same amino acid alteration (PS1); *de novo* occurrence (PS2) ;location in functional domains(PM1);the allele frequency in population databases (gnomAD v4.1.1) is extremely low(PM2);multiple in silico prediction tools predict a deleterious effect(PP3); ClinVar databases indicate pathogenic(PP5). But in Clinvar and Franklin databases, this variant is classified as pathogenic. According to the latest 2024 IUIS (International Union of Immunological Societies) classification of IEIs (Inborn Errors of Immunity), *STAT3* GOF is categorized as a disease of immune dysregulation (25).

## 3 Conclusion

### 3.1 Computer simulation prediction

**Figure 3:**
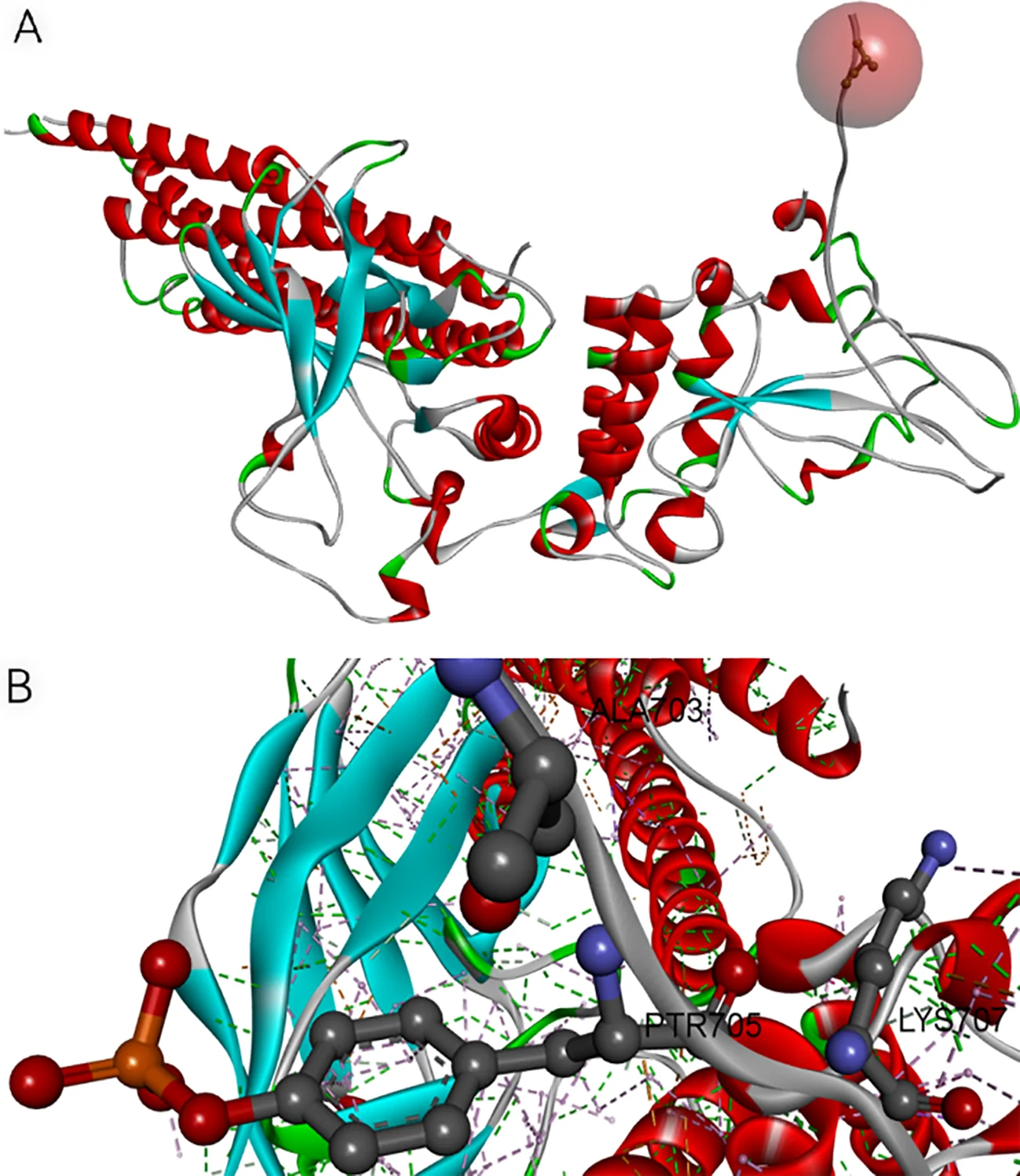
Three-dimensional model of STAT3 **(A)** Residue Ala703 is highlighted with a red circle. **(B)** Close-up view of the Ala703 subregion in STAT3 (ribbon diagram). Phosphorylated tyrosine residue is represented as Ptr705, Lys707 residue is shown in grayish-blue, and hydrogen bonds are depicted as green dashed lines.

The impact of the Ala703Thr mutation on *STAT3* was investigated by constructing a 3D model of *STAT3* using Discovery Studio, based on *STAT3* structures available in the Protein Data Bank (PDB codes: 6QHD, 4E68, 6NJS, 6UNQ). All experimental structures were determined by X-ray crystallography with good quality and resolutions ranging from 2.25 Å to 3.15 Å. This model allowed comparison between the wild-type and mutant proteins, with a focus on the region surrounding residue Ala703. In native *STAT3*, Ala703 is located near Tyr705 and forms three hydrogen bonds with the main chain of Lys707, similar to Ala705. Like almost all alanine residues in *STAT3*, the essential side chain of Ala703 plays a key role in establishing tighter interactions within specific protein subregions to ensure proper protein folding, according to the model. The lack of the essential alanine group on the main and side chains of phosphorylated tyrosine 705 negatively affects the extensive hydrogen bond network around residue 703. The substitution of alanine at position 703 with threonine fails to participate in all the hydrogen bonding interactions facilitated by alanine. Since tyrosine 705 is the primary site for dimerization following *STAT3* tyrosine protein phosphorylation, upon phosphorylation of Tyr705, its main and side chains form hydrogen bonds with amino acids such as Arg609 in the SH2 domain of another STAT3 monomer to mediate dimerization. The missense mutation from alanine to threonine at residue 703 leads to a loose conformation in the main chain hydrogen bond region among Ala703, Tyr705, and Lys707, indirectly affecting the phosphorylation of Y705 and the stability of the SH2 binding pocket. This may be associated with *STAT3* dysfunction, such as the development of autoimmune diseases. Therefore, according to the 3D model, the mutation adversely affects protein folding and also negatively impacts subsequent *STAT3* dimerization, indicating structural support for a GOF mechanism (**Figure 3**). Whether it results in enhanced activity of *STAT3* monomers, delayed or impaired *STAT3* dephosphorylation, increased stability of aberrantly formed *STAT3* dimers, or enhanced affinity for DNA targets are downstream mechanisms that urgently require further investigation (21, 26–32).

### 3.2 Comprehensive immunological examination and assessment

**Figure 4:**
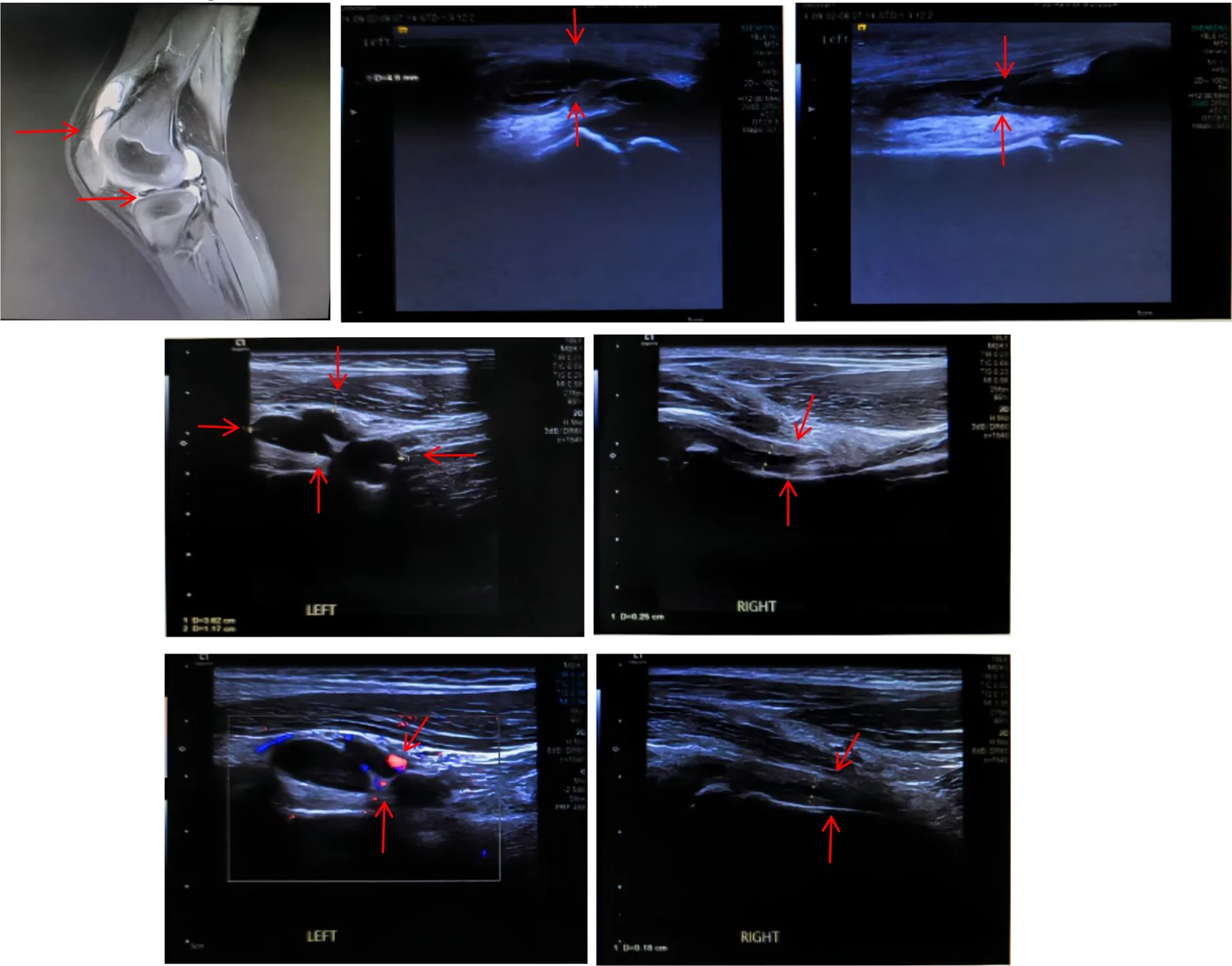
Sep.15.2024 Osag fs T1WI left: Effusion in left knee joint and suprapatellar bursa, accompanied by synovial thickening and synovitis (4). Sep.2 Synovial thickening of the left knee joint, increased blood flow (Grade I), and fluid depth of approximately 0.5cm in the suprapatellar bursa (5,6) May 28.2025 No evidence of effusion in the left hip joint; fluid depth of approximately 0.2cm in the right hip joint (7,8) July 17.2025 No evidence of effusion in the left hip joint;fluid depth of approximately 0.3cm in the right hip joint (9,10).

The patient’s later-onset joint symptoms were not isolated but were closely related to his initial symptoms. He developed the disease during infancy. Although multiple early symptoms did not appear simultaneously with the *STAT3* GOF gene mutation, they progressively affected the patient’s knee and ankle joints (**Figure 4**), lungs, as well as the cervical and inguinal lymph nodes over the course of the disease. Therefore, we should evaluate his symptoms comprehensively and in relation to one another, rather than separately. Thus, ADMIO type 1 disease resulting from the *STAT3* GOF mutation is considered (33–35).

The evidence supporting the pathogenicity of our variant comes from the whole-exome sequencing report and the sequential appearance of typical phenotypes, which certainly also contributed to the delayed diagnosis in our case. Since the patient’s clinical manifestations are consistent with ADMIO type 1, according to the literature, a *de novo* mutation may also represent an autosomal dominant inheritance pattern. Furthermore, although whole-exome sequencing did not identify a gain-of-function (GOF) mutation pattern, we cannot definitively exclude the presence of intronic variants, as the entire gene was not sequenced.

**Figure 5:**
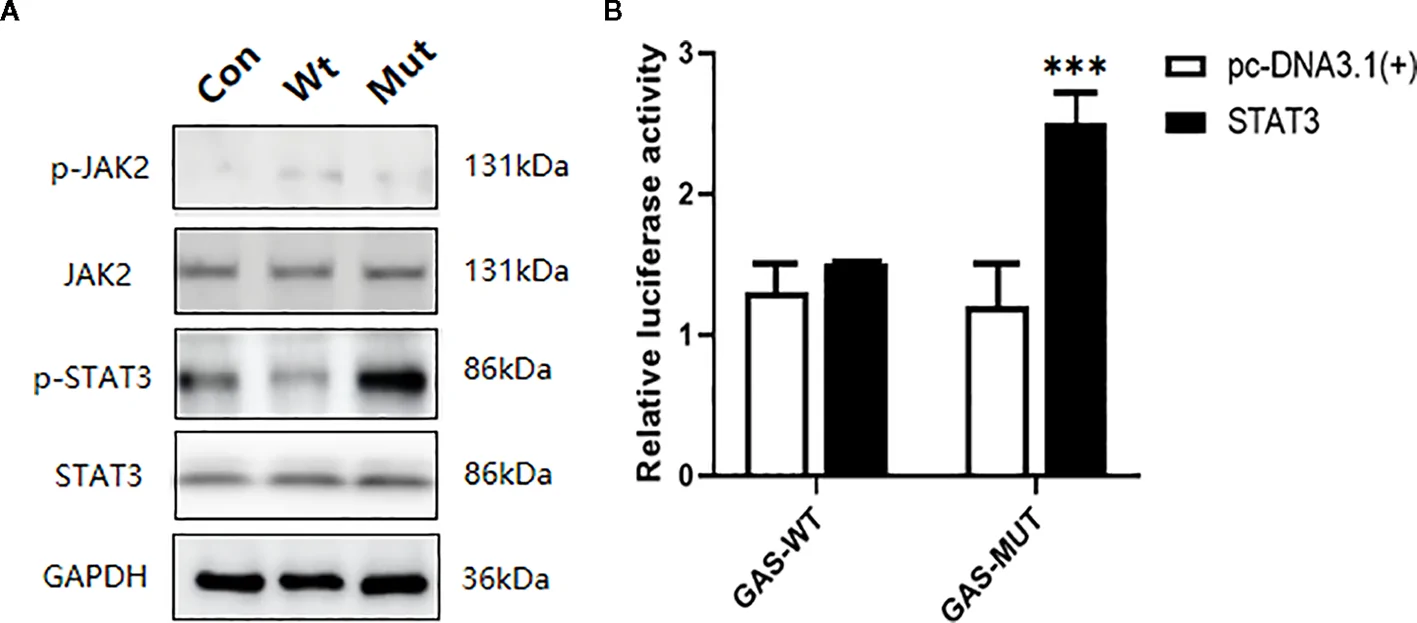
Vitro functional analysis of STAT3 mutants. **(A)** Protein expression of the JAK-STAT3 pathway. **(B)** Relative luciferase activity of GAS-WT and GAS-MUT following transfection with wild-type or mutant STAT3 plasmids.

Western blot analysis revealed enhanced protein expression following the mutation at this site in the patient, which is opposite to the typical loss-of-function (LOF) mutation pattern characteristic of *STAT3* LOF mutations. For this variant, western blot assay suggested that mutant protein exhibits elevated expression levels. Enzyme activity experiment demonstrated that the enzymatic activity of the mutant was significantly higher than that of the wild-type, resulting in sustained downstream phosphorylation and continuous activation, thus confirming a gain of function. Additionally, reduced protein degradation, prolonged half-life, and increased expression levels of the mutant protein further support a gain-of-function effect. Regarding signaling pathway activity, experiments demonstrated hyperactivation of downstream pathways (p-JAK2, p-STAT3, p-ERK, p-AKT), indicating sustained pathway activation, which is a typical manifestation of GOF. CCK8—cellular functional assays confirmed that the mutant cells exhibited increased proliferation, decreased apoptosis, enhanced migration, and abnormal differentiation, characterized by overgrowth and overactivation, which consistents with the gain-of-function phenotype. In the patient, in silico studies indicated that A703T mutation is located within a critical functional domain (the SH2 domain), and substitution of alanine at position 703 with threonine prevents normal *STAT3* tyrosine site dimerization, thereby activating downstream signaling and impairing proper termination, leading to aberrant downstream activation through disruption of the spatial structure. Dual-luciferase reporter assay results revealed that the mutant GAS-MUT exhibited higher luciferase activity than GAS-WT, indicating that the GAS-MUT construct unexpectedly conferred a significantly enhanced transcriptional activation capacity. This suggests that the GAS-MUT functions as a GOF mutant in the context of STAT3-mediated transcriptional regulation (**Figure 5**). Finally, regarding the inheritance pattern, autosomal dominant inheritance is the most typical mode of transmission for GOF mutations, where heterozygosity alone is sufficient to cause disease.

In ADMIO type 1, joint MRI typically shows sterile arthritis and synovial thickening. Similar arthritis-associated autoimmune diseases caused by analogous gene mutations have been observed in other autosomal dominant disorders (ADMIO2 caused by ZAP70 gene mutation), consistent with our findings (36). Although the symptoms lack specificity, involvement of the immune system and the osteoarticular system is also a common manifestation of ADMIO type 1. In addition, rare cases of interstitial lung disease, diabetes mellitus (37), and lymphoma associated with ADMIO type 1 have been reported.

With the increasing use of NGS technologies, detection of STAT3 heterozygous mutations may occur even in asymptomatic patients with ADMIO type 1. In cases with a mild phenotype, after excluding duplications/deletions of other alleles and confirming that the variant affects protein structure or function, a diagnosis of *STAT3* GOF may be considered.

## 4 Discussion

**Figure 6:**
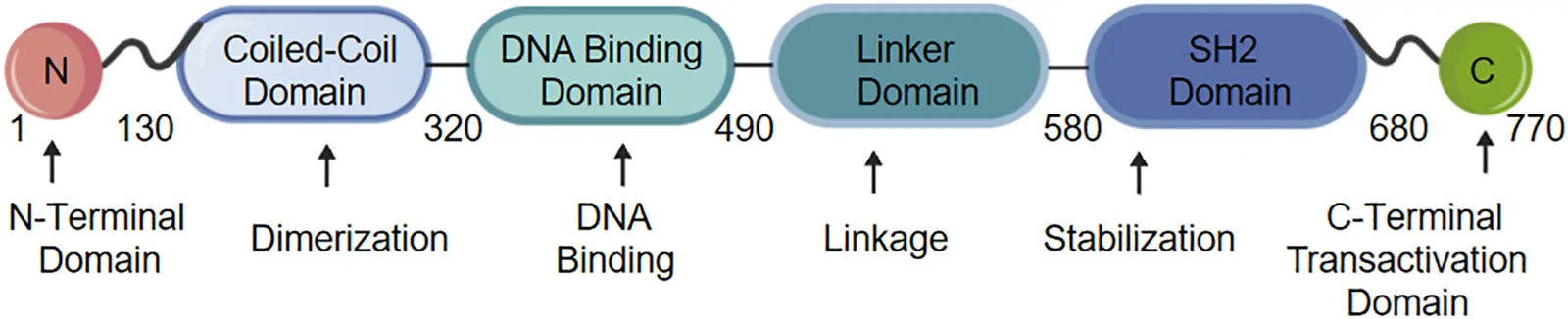
Domain of STAT3 gene.

**Figure 7:**
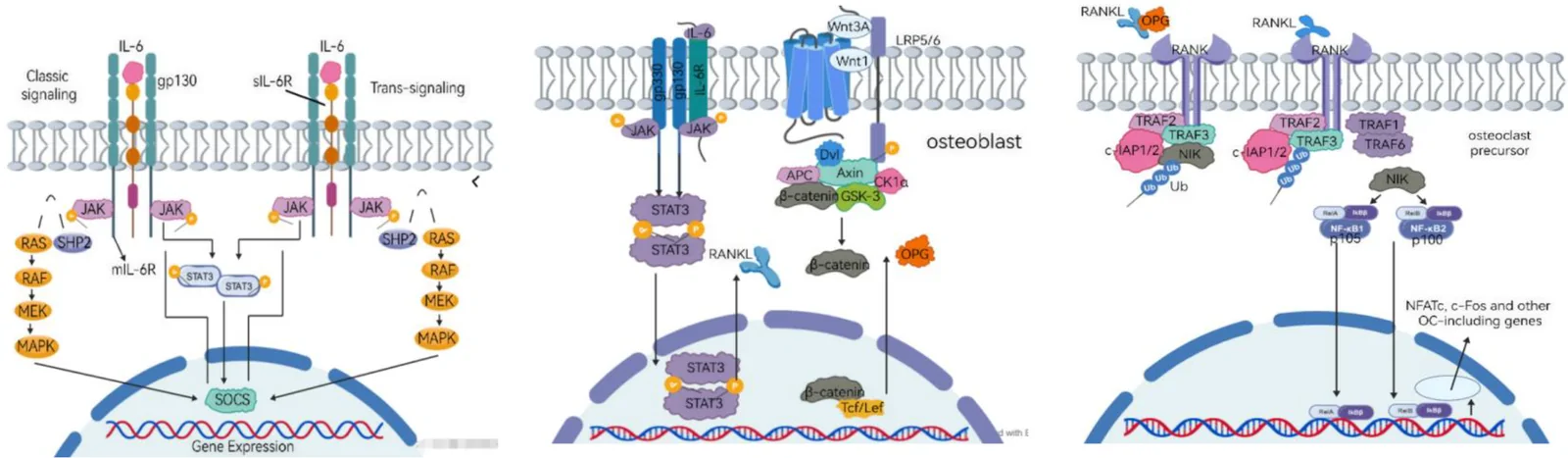
Mechanism of bone destruction.

*STAT3* encodes a key *STAT3* protein in the JAK-STAT3 signaling pathway, which is a member of the *STAT* protein family. Each STAT protein contains six major domains: the N-terminal domain, coiled-coil domain, DNA-binding domain, linker domain, SH2 domain, and transactivation domain (**Figure 6**). These domains collaborate to enable *STAT* proteins to perform complex signal transduction functions. Among them, the SH2 domain is the most critical of all domains. Its main function is to recognize and bind to specific tyrosine residues phosphorylated by JAK kinases (8), thereby creating conditions for subsequent phosphorylation of *STAT* proteins. Although the exact pathogenic mechanism of *STAT3* mutations in ADMIO type 1 remains not fully understood, it appears to involve multiple abnormal functions, including excessive activation of downstream *STAT3* signaling pathways. Nearly all are missense mutations, with no clear genotype-phenotype correlation established (1, 5, 8, 9, 11, 15, 18, 20, 21, 32, 38–43). Under pathological conditions, cytokines such as those of the IL-6 family bind to their receptors, inducing gp130 dimerization and subsequent JAK activation. Activated JAKs recruit *STAT3*, leading to its phosphorylation at the Y705 site. Through interactions involving the SH2 domain, phosphorylated *STAT3* dimerizes and translocate to the nucleus, where it acts as a transcription factor to regulate gene expression, including the expression of RANKL, thereby influencing the maturation and differentiation of osteoblasts (OBs). Upon activation, the expression of Receptor Activator of Nuclear Factor-κB Ligand (RANKL) is significantly upregulated, while the expression of osteoprotegerin (OPG) is downregulated. RANKL binds to the Receptor Activator of Nuclear Factor-κB(RANK) receptor on the surface of osteoclast precursor cells, activating the NF-κB signaling pathway and thereby promoting the differentiation of osteoclast (OC) precursors into mature OCs. Meanwhile, the Wnt pathway is activated and mediates the downregulation of OPG expression, with OPG acting as a decoy receptor to inhibit this process. Pro-inflammatory cytokines (IL-6, IL-17, TNF-α) and hypoxia-inducible factor-1α (HIF-1α) can promote bone resorption by upregulating RANKL (**Figure 7**) (44).

The reasons why the patient was ultimately suitable for treatment with ruxolitinib (45) are as follows: the core mechanism is by inhibiting JAK1/JAK2 kinases, which blocks upstream JAK/STAT signaling, thereby suppressing the persistent activation of mutant *STAT3* and downstream abnormal transcription, ultimately correcting immune imbalance and inflammation. Effects on *STAT3* GOF show it blocks cytokine signals that drive *STAT3* activation, including IL-6, IL-21, IL-23, and IFN-γ, reducing p-STAT3 (Y705) levels, inhibits persistent nuclear translocation and transcriptional activity of mutant *STAT3* and downregulate *STAT3* target genes (Socs3, Maf, Cxcr3) as well as interferon-related genes, myeloid activation genes, and cytotoxic CD8+ T cell genes. Based on the above, IL-6 inhibitors provide upstream blockade with a single target and are prone to treatment failure; tofacitinib inhibits JAK3/JAK1, offering moderate suppression of *STAT3*; TNF-α monoclonal antibodies do not directly target *STAT3* and only control severe inflammation; ruxolitinib potently inhibits JAK1/JAK2, providing the most direct targeting of *STAT3* GOF, particularly for GOF mutations in the *STAT3* TAD domain. Therefore, it is most suitable for diseases characterized by persistent *STAT3* activation, which targets upstream of *STAT3* more effectively than tofacitinib (46) and is more efficacious for lymphadenitis, arthritis, pneumonia, and enteritis.

Currently, there are no definitive diagnostic criteria or precise treatment protocols for ADMIO type 1. Genetic testing, when combined with a comprehensive clinical assessment, is the only reliable method to confirm the diagnosis. Treatment (47–49) typically involves the use of immunosuppressants, hormone replacement therapy, anti-infective agents, and biologics (19). Literature indicates that 85% of diagnosed children exhibit clinical involvement of at least three different systems, often necessitating combination therapy. Although glucocorticoids are the first-line treatment, they are effective in only a minority of patients. Most pediatric patients require combination therapy with multiple immunosuppressants, and nearly half of them need three or more drugs simultaneously. This is one of the few reports describing the heterozygous *STAT3* GOF c.2107G>A, p.Ala703Thr variant as a potential cause of ADMIO type 1. According to in silico studies, the substitution of alanine at position 703 with threonine is predicted to interfere with protein folding. Notably, whole-exome sequencing revealed only the *STAT3* c.2107G>A (p.Ala703Thr) mutation as a potential explanation for the patient’s secondary arthritis. Furthermore, combining the clinical manifestations, ultrasound findings, arthrocentesis results, and in silico studies strongly supports vasculitis and arthropathy as the underlying causes. However, partial in silico and preliminary experimental verification of the STAT3 gain-of-function variant was completed under limited conditions. Further large-sample research is needed to generalize its common clinical features and explore pathogenic mechanisms.

## Data Availability

The original contributions presented in the study are included in the article/supplementary material, further inquiries can be directed to the corresponding author/s.
